# Heterogeneity of Subcellular Diffusion in Bacteria Based on Spatial Segregation of Ribosomes and Nucleoids

**DOI:** 10.1159/000526846

**Published:** 2022-09-07

**Authors:** Simon Dersch, Daniel A.O. Rotter, Peter L. Graumann

**Affiliations:** Centre for Synthetic Microbiology (SYNMIKRO) and Fachbereich Chemie, Philipps-Universität Marburg, Marburg, Germany

**Keywords:** Subcellular architecture, Chromosome arrangement, Translation, Protein diffusion, Single molecule tracking

## Abstract

It has long become clear that in spite of generally lacking internal membrane systems, bacteria contain well-structured subcellular structures of usually filamentous proteins, and a preferred 3D arrangement of their chromosome(s). Some of these systems are set up by so-called cytoskeletal elements, or by polar landmark proteins, but the mechanism of specific localization is still unclear in most cases. Intriguingly, apart from such spatially organizing systems, the bacterial cytoplasm has unusual properties in terms of the diffusion of molecules, which varies between different sites within the cell. In many bacteria, chromosomes are compacted into centrally located nucleoids, being orderly folded as opposed to consisting of random coils of DNA. In these bacteria, there is a separation of transcription and translation, such that transcription by RNA polymerase occurs on the nucleoids, and translation takes place mostly at the cell poles and directly underneath the cell membrane, because 70S ribosomes accumulate at sites surrounding the nucleoids. Interestingly, accumulation of ribosomes appears to slow down diffusion of enzymes, noticeable for larger enzyme complexes, while nucleoids provide areas of confined motion for DNA-binding proteins, yet acceleration zones for non-DNA-binding proteins. Crowded regions at the cell poles set up zones of higher concentration of the translation machinery, shortening diffusion distances for rate-limiting translation factor/ribosome interactions, and of metabolic enzymes, possibly speeding up pathways containing low concentrations of metabolites. Thus, heterogeneous diffusion adds another layer of subcellular organization on top of cytoskeletal elements.

## Subcellular Organization in Bacterial Cells

Bacteria have evolved a variety of proteins that can assemble into mostly filamentous structures to organize different features of cells, be it the shape of the cell wall, cell division, localization of protein complexes, or DNA replication [Surovtsev and Jacobs-Wagner, 2018]. Self-organized protein filaments or meshes can provide directionality or positional information, most notably used by proteins that assembly at the cell poles in rod-shaped bacteria and recruit other proteins or chromosome sites to these subcellular regions [Rudner and Losick, 2010; Linet al., 2017]. This review deals with biophysical aspects of proteins, with regard to their diffusion properties, which are employed by bacterial cells to organize several physiological aspects of prokaryotic life. We will discuss that: (a) chromosomes have a preferred 3D arrangement within bacterial cells, (b) many bacteria contain compacted nucleoids and translating ribosomes at sites surrounding the nucleoids, (c) accumulation of ribosomes at mostly polar spaces in cells set up areas of high protein crowding affecting the diffusion of even freely diffusing enzymes, and (d) that the preferred positioning of ribosomal RNA operons at the polar sites of the nucleoids, directly adjacent to ribosome-containing polar sites, sets up a nucleolus-like arrangement in which rRNA is directly loaded with ribosomal proteins at subpolar sites. We will argue that bacterial cells contain a layer of complexity, based on heterogeneous diffusion of proteins, that was not appreciated until very recently.

**Fig. 1:**
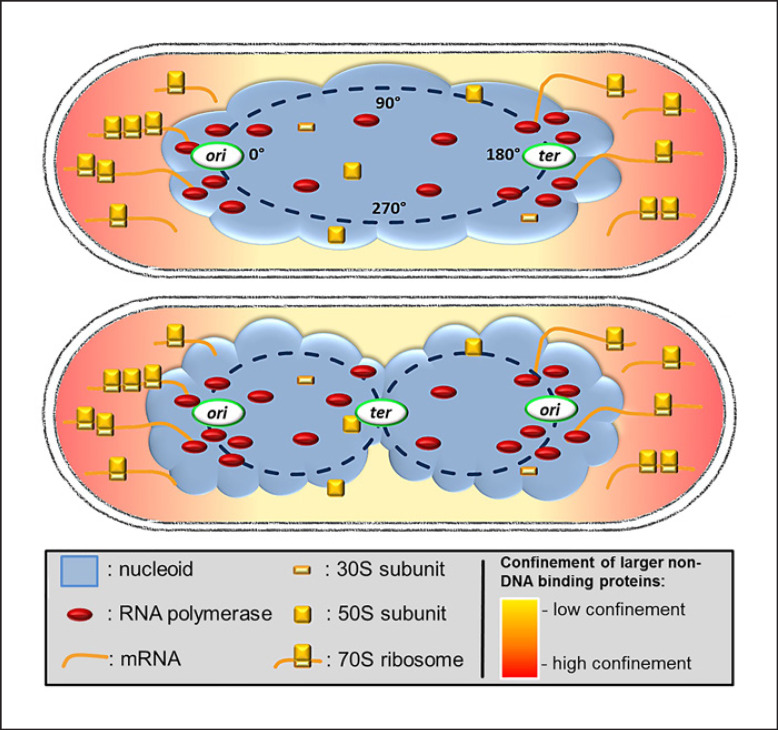
Model for the arrangement of a nucleoid-containing, rod-shaped bacterial cell. Many bacteria contain a cloud-like structure, the nucleoid, which contains an in itself helical structure of the compacted chromosome(s) (note that many bacteria are polyploid [Soppa, 2021]), likely featuring toroidal loops of DNA emanating from a general linear arrangement of chromosome sites from one end of the nucleoid to the other, as indicated in the upper cell. The lower cell represents a replicating cell, where origin regions have segregated toward opposite outer edges of nucleoids.

Many bacterial cells contain a compacted structure of their chromosome(s) called the nucleoid, which splits into two nucleoids before cell division (Fig. 1). We will deal with these bacteria including many model organisms such as *Escherichia coli* and *Bacillus subtilis* and elaborate that the existence of the centrally located nucleoids sets up a spatial separation of transcription and translation, which in turn also affects general protein mobility within bacterial cells. Note that the alpha-proteobacterium *Caulobacter crescentus* contains its DNA throughout the cytosol and appears to organize transcription and translation in spatially overlapping manner, which will be described further below. For the purpose of this review, we are ignoring the phylum “Planctomycetes,” where many members possess very unusual intracellular features [Wiegand et al., 2018].

Within the nucleoids (or within the entire cell in case of *C*. *crescentus* and other alpha-proteobacteria), chromosomes have a preferred layout of the chromosome: at the onset of the cell cycle, the origin region (region of initiation of replication) is located at one edge of the nucleoids, and the terminus region at the other edge (Fig. 1). The chromosome is compacted according to its ring shape, such that regions from origin toward terminus are also positioned in this order along the length of the nucleoid, and chromosome arms are juxtaposed [Graumann, 2014]. After initiation of replication, one duplicated origin moves across the nucleoids toward the other cell pole, and the terminus travels toward the cell center, where it is replicated last in the cell cycle (Fig. 1). The cell divides with two completely separated nucleoids, in which the preferred chromosome arrangement is retained. This simplified description shows variations between different bacterial species, and during rapid growth, arrangement of origin and terminus regions becomes more complex than shown in Figure 1. Chromosome architecture is not static: DNA segregation demands large distances of motion of duplicated chromosome regions, and additionally, chromosome sites show constrained diffusive motion in the range of seconds at any time in the cell cycle [Javer et al., 2013; Schibany et al., 2018]. Clearly, chromosome segregation and chromosome compaction are well-ordered processes in bacterial cells.

## Spatial Separation of Translation and Transcription in Bacteria Containing Nucleoids

In 2000, the bacterial cell biology field was taken by surprise when the Errington group showed that a fluorescent protein fusion of the ribosome localized outside the nucleoid, indicating that translation occurs at sites surrounding the cellular DNA, while RNA polymerase (RNAP) localized on the nucleoids [Lewis et al., 2000]. A year later, it was shown that the occlusion of ribosomes from nucleoids depends on active transcription [Mascarenhas et al., 2001], suggesting that mRNA synthesized on the nucleoids is expelled from this space and translated at surrounding sites, such that ribosomes accumulate mostly at the poles dependent on their substrate (Fig. 1). The 2000 study also revealed that during rapid growth, where replication is driven by multiple replication forks on the chromosomes, RNAP accumulates at the outer edges of the nucleoids where origin regions of chromosomes are located for most of the cell cycle [Lewis et al., 2000]. Super resolution fluorescence microscopy showed that the accumulation of RNAP at outer edges of the nucleoids depends on active transcription and not on the density or amount of DNA, i.e., transcription can relocate chromosome regions to the nucleoid periphery and lead to RNAP clusters [Stracy et al., 2015]. High rates of transcription correlate with the fact that half or more rRNA operons in, e.g., *E*. *coli* or *B*. *subtilis* are located close to origin regions, besides many highly transcribed genes. Indeed, even rRNA operons distant from origin regions cluster with those close to origin regions [Gaal et al., 2016]. Because of the proximity of these so-called transcription foci or TFs to the polar regions containing 70S ribosomes, it was proposed that many bacteria have a nucleolus-like structure, where newly synthesized rRNA needs to diffuse a very short distance until newly synthesized ribosomal proteins can be added, or in other words, a spatial coupling between rRNA synthesis and ribosome assembly [Gaal et al., 2016; Jin et al., 2017]. However, it has been shown that free ribosomal subunits can diffuse through the entire cell, while only 70S ribosomes and polysomes are excluded from the nucleoids [Sanamrad et al., 2014], such that subunit assembly may also be a freely diffusive process. We will pick up this idea later on.

The general separation between transcription and translation in many bacteria does not contradict the mechanism of transcriptional attenuation such as in *E*. *coli*, where efficiently translating ribosomes determine termination or antitermination of RNAP, in other words, where transcription and translation are temporally directly coupled; several studies showed that there is a spatial overlap of RNAP and translating ribosomes, in the order of 10–15% of ribosomes overlapping with the chromosome and thus with RNAP molecules [Bakshi et al., 2012]. In contrast to this scenario, the Jacobs-Wagner group has shown that in *C*. *crescentus*, sites of translation of proteins can be identified in 2D and generally coincide with the position of the corresponding gene locus [Montero Llopis et al., 2010]. As explained above, bacterial chromosomes have a relatively defined 3D arrangement inside the cell, where any gene locus has a preferred subcellular location [Graumann, 2014]. Thus, in *Caulobacter* cells, ribosomes translate mRNA close to the site of their synthesis, while in nucleoid-containing bacteria, mRNA and other RNA molecules need to diffuse out of the nucleoid to be translated outside of this area [Bakshi et al., 2012]. A recent study indeed suggests that mRNAs can diffuse through the entire cell in few seconds, such that principally, they can reach any position in the cell within very short time [Sattler and Graumann, 2021].

## Self-Organization of Bacterial Cells Using Entropic Forces

Bacterial chromosomes are generally about 2,000 times larger than the cell when completely extended into a circle. One level of compaction is achieved by topoisomerases that produce supercoils by over-twisting − or usually under-twisting − of the DNA helix [Graumann, 2014]. It has been proposed that thermal collisions between DNA and the large number of soluble proteins in cells provides a compaction energy that overwhelms the expansion energy of DNA strands, causing a phase separation between the DNA and the surrounding cytoplasm [Woldringh and Nanninga, 2006]. Another factor of chromosomal positioning in bacterial cells are entropic effects, which likely govern the general organization of cellular components. Modeling studies have suggested that in a minimalistic system, the compacted DNA macromolecule would generally avoid the polar regions to maximize entropy, while placement of ribosomes would entropically favor the less occupied peripheral regions [Mondal et al., 2011]. Dynamics simulations additionally showed that, in cells with a compacted nucleoid, mRNA molecules would also tend to localize predominantly in the polar regions, due to volume exclusion effects [Castellana et al., 2016]. Computational models also show that segregation of chromosomes during division may arise from spontaneous, entropy-driven de-mixing of daughter strands under certain conditions [Jun and Wright, 2010], and in vivo studies showing patterns of directional diffusion of duplicated chromosome regions have provided strong support for the idea of entropic repulsion of DNA strands providing a major force for segregation [El Najjar et al., 2020]. It is thus an important concept to realize that fundamental physical forces may provide a reasonable explanation of several aspects of cellular self-organization.

Protein mobility in bacterial cells is highly affected by physical properties of cells, e.g., by changes in macromolecular crowding in response to osmotic upshift in the medium [van den Bogaart et al., 2007; Konopkaet al., 2009]. How far factors such as internal pressure or temperature affect chromosome dynamics or self-organization of bacterial cells is still understudied.

## Nucleoids and Chromosomes Providing a Grid for Confined Motion of DNA-Binding Proteins

How to find a DNA-binding site? It has long been thought that the condensed nucleoid, containing a multitude of proteins plus compacted DNA, should provide a diffusion barrier for proteins. For DNA-binding proteins on the other hand, free diffusion alone, plus a proposed sieving effect of the nucleoids, cannot account for the required efficiency of the target search process, unless the protein is highly abundant. Unspecific DNA binding, or one-dimensional motion along what is essentially a DNA “highway” could contribute to increasing the rate at which specific proteins are able to find target sequences, by largely confining them to DNA-rich regions of the cell. This has been theorized by a multitude of studies for a subset of DNA binding proteins, and the implications of the underlying mechanisms have been comprehensively summarized by Halford and Marko [Halford and Marko, 2004].

Looking at various studies, DNA-binding proteins are indeed largely found to localize on the nucleoids, or even to specific subregions of the nucleoids. Nonspecific DNA binding will generate hopping from one DNA strand to the next, leading to constrained diffusion of proteins as opposed to free Brownian motion. Of course, any DNA-binding protein will also show a degree of free diffusion within nucleoid-containing bacteria, when it leaves the nucleoid areas. Motion of DNA-binding proteins has been quantified by single molecule tracking. A dimeric Lac repressor (LacI, usually forms tetramers when bound to DNA) requires about 6 min for finding its specific *lacO* site, and it does so by interacting nonspecifically with the DNA for 90% of its lifetime [Elf et al., 2007]. Nonspecifically bound LacI diffuses along DNA with a residence time of <5 milliseconds [Elf et al., 2007], as opposed to a dwell time of 5 min for a *lacO* site [Hammar et al., 2014]. On the other hand, a dCas9 protein needs 6 h to find a single guide-RNA-directed site in the *E*. *coli* chromosome [Jones et al., 2017], and a CASCADE complex 1.5 h, whereby it spends about half of its time freely diffusing and 50% hopping through the chromosome [Vink et al., 2020]. Thus, cells require a relatively large number of CRISPR/Cas complexes to efficiently detect newly incoming phage DNA. Binding kinetics can also vary drastically, with CRISPR/Cas systems being bound to DNA (including opening strands and probing for homology to guide RNA) for 30 ms on average [Vink et al., 2020], while for some DNA-binding proteins, nucleoid association is very strong. An example for this is structural maintenance of chromosomes (Smc) protein, which shows either tight binding to DNA for minutes (probably molecules engaged in ATP-driven DNA loop extrusion) or constrained diffusion through the nucleoid, with freely diffusing molecules being hard to detect [Kleine Borgmann et al., 2013; Schibany et al., 2018].

For a restriction endonuclease/methylase system, it has been shown that the methylase is mostly found as diffusing through the nucleoids in a constrained manner or being tightly bound, while for the endonuclease, a majority of molecules were freely diffusive, suggesting that the restriction endonuclease system largely finds DNA-binding sites through direct hits, or short events of moving along DNA strands [Negri et al., 2021].

Recently, by utilizing single molecule tracking techniques, Stracy et al. [Stracy et al., 2021] have addressed the question if nonspecific DNA interactions are a general contributor to target search of DNA-binding proteins. As expected, motion of DNA-binding proteins is largely governed by specific as well as nonspecific interactions with DNA. Surprisingly, their diffusion constants only mildly depend on their size, such that variations in dynamics refer to how much time they spend hopping through the nucleoid, or in other words, how many molecules are nucleoid associated or freely diffusive. This ratio can vary between 50 and 99% [Stracy et al., 2021]. Thus, target search is largely governed by sliding or hopping through the chromosome, via transient nonspecific interactions, where the nucleoids seemingly perform a nucleus-like function in keeping most DNA-binding proteins localized centrally, while they search for their preferred binding site(s). On the other hand, the mobile fractions of even larger DNA-binding proteins seem to be unaffected by the nucleoid, with regards to their diffusion [Stracy et al., 2021].

Taken together, this strong enrichment of DNA-binding proteins directly at the nucleoid suggests that this region is densely populated but does not generally expel larger non-DNA-binding proteins or assemblies; however, accumulation of the highly abundant translating ribosomes bound to mRNA at sites surrounding nucleoids, as well as of other proteins (see below), the phenomenon of nucleoid occlusion (NO, Fig. 1) gives rise to the formation of what could essentially be termed a pseudo-nucleus.

## Accumulation of Ribosomes Leads to Areas of Confined Motion of Enzymes at the Cell Poles

It has been a long time since bacteria have been considered single cells lacking any particular internal structure other than randomly diffusing enzymes. However, in addition to a variety of proteins that assemble into polymeric structures at specific sites in cells, or proteins that recognize subcellular cues and assemble, e.g., specifically at highly curved cell poles in order to provide a landmark for the recruitment of many other proteins [Lam et al., 2006; Rossmann et al., 2019], it is slowly becoming clear that bacteria contain areas of different degrees of protein crowding that may help organize several subcellular processes. The mentioned study on the motion of DNA-binding proteins through normal and artificially DNA-free *E*. *coli* cells also revealed that the nucleoids are not physical barriers for free protein diffusion [Stracy et al., 2021]. Computational modeling has suggested that the accumulation of ribosomes at the cell poles will lead to a generally higher concentration of proteins due to molecular crowding [Trovato and Tozzini, 2014].

**Fig. 2:**
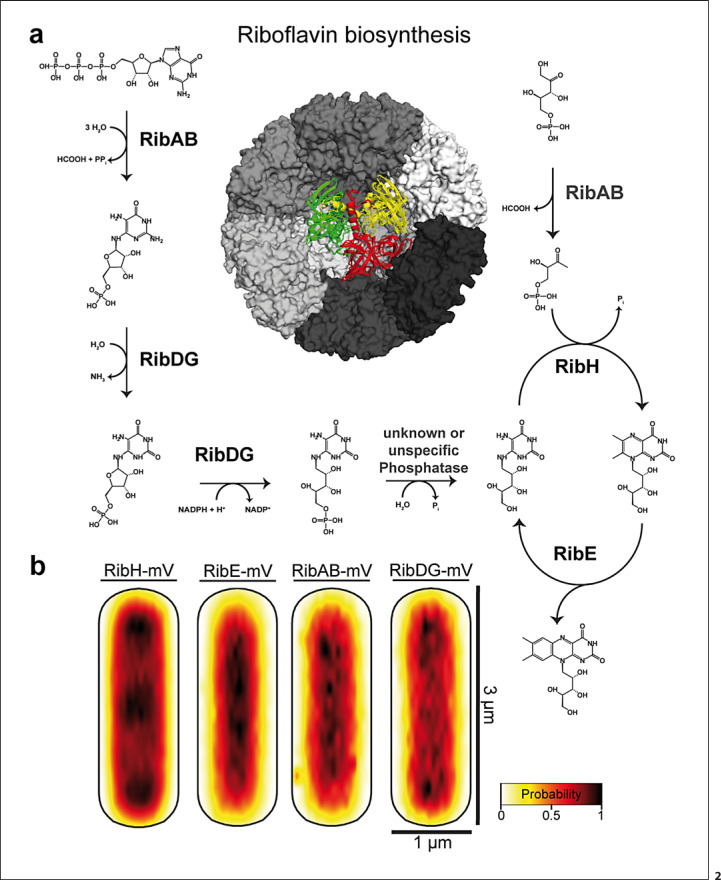
**a** Pathway of riboflavin synthesis, starting from GTP and ribulose 5 phosphate. Heavy riboflavin synthase consisting of RibE and RibH is shown in the center − the RibE trimer is projected onto the center − but in reality is encased within the 60mer RibH structure. **b** Heat maps showing all tracks of enzymes (mV = mVenus fusions) projected into a medium-sized *Bacillus subtilis* cell. RibH and RibDG show depletion of molecules from the cell center, so-called “nucleoid-occlusion” (NO). Accumulation of RibH at the poles occurs throughout the cell cycle, while accumulation in the cell middle occurs only in large cells [Rotter et al., 2021].

**Fig. 3:**
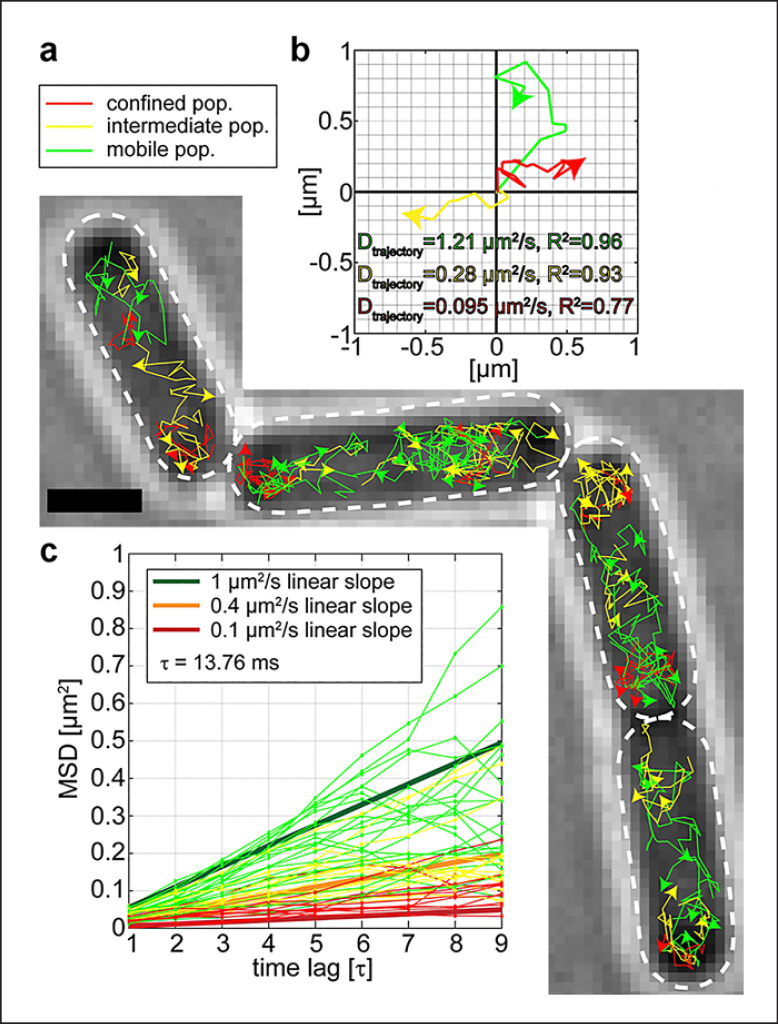
Single molecule tracking of RibH-mVenus reveals different modes of diffusion of the same molecule in *Bacillus subtilis* cells. **a** Trajectories showing low diffusion constants due to confined motion are shown in red, those showing intermediate diffusion in yellow, and those showing free diffusion in green. **b** Mean squared displacement of tracks sorted into three populations. **c** Examples of each type of trajectory in a coordinate system.

In line with this idea, a large protein complex essential for riboflavin (RF) biosynthesis was found to accumulate at the cell pole areas in *B*. *subtilis*, the RF synthase complex [Rotter et al., 2021]. The first steps of RF biosynthesis are carried out by RibAB and RibDG, while the final steps are catalyzed by a large complex consisting of 60-mer RibH capsid containing homotrimeric RibE, termed heavy RF synthase (Fig. 2a). This structure generates substrate channeling because one product of RibE is a substrate for RibH, whose product is again a substrate for RibE (Fig. 2a). In spite of channeling, only 14% of RibE are encapsulated by RibH, while the bulk of the protein is mostly freely diffusive within the cytoplasm, meaning the final steps of biosynthesis can also occur in a decoupled (i.e., freely diffusive) manner [Rotter et al., 2021]. Using a single-molecule tracking approach, it was found that the heavy RF synthase, visualized via a RibH-mVenus fusion, is occluded from the nucleoids, similarly to ribosomes (Fig. 2b). While RibAB and free RibE did not show NO, the large RibDG enzyme did (Fig. 2b). When molecules were sorted into populations having distinct diffusion constants, RibAB and RibDG generally showed the highest diffusion in the center of the cytoplasm, i.e., the nucleoid; contrarily, the peripheric regions and cell poles are zones where they tend to diffuse at a lower rate. RibH/the heavy RF synthase showed a similar behavior, with the most pronounced degree of slowing down in polar regions [Rotter et al., 2021]. Thus, areas of slow diffusion coincide with the areas of highest localization probability, effectively depleting especially the large enzymes from nucleoid areas. Figure 3 shows an example of SMT analysis: RibH molecules can be sorted into three categories: those that show very low mobility (red trajectories, Fig. 3b), those that show intermediate mobility (yellow trajectories), and a population of fast-diffusing molecules (green trajectories). Slow- and medium-diffusing molecules are mostly found close to the cell poles (Fig. 3a), and fast-diffusing tracks mostly away from the poles. Figure 3c shows examples for tracks. More granular analysis of the different subpopulations of diffusion revealed that the slower RibH molecules show strongly confined motion at the poles, which manifests itself as a pattern of NO in localization probability maps (Fig. 2b). The smaller enzymes RibE and RibAB, on the other hand, showed no strong NO pattern, supporting the idea that the molecular crowding in the polar regions appears to mainly affect larger protein assemblies. Furthermore, after arrest of active transcription via rifampicin treatment, the NO effect for RibH was strongly reduced, suggesting that active ribosomes play a key role in setting up a micro-environment that confines other large molecules [Rotter et al., 2021].

Taken together, it appears that fast diffusion of non-DNA-binding proteins occurs predominantly across the center of the cell because the nucleoid does not provide a diffusion barrier for proteins, while lower, confined diffusion is localized to the crowded cell poles, due to accumulation of ribosomes in these regions. This behavior, if indeed a general property, could help facilitate the acceleration of cell metabolism, especially for low-metabolite pathways, where metabolites rapidly diffuse from one enzyme to the next.

## A Nucleolus-Like Organization in Bacteria

Diffusion constants of some cytoplasmic proteins in *E*. *coli* ranging from 26 kDa to 70 kDa have been shown to scale with the square of their radii, whereas proteins ranging from 70 kDa to 250 kDa revealed only a small size dependence and reached a plateau at 0.8 µm^2^/s [Kumar et al., 2010]. A study using phage particles in *C*. *crecentus* cells found a similar upper limit for diffusion, independent of particle size, and interestingly showed that protein mobility is higher in metabolically active than in inactive cells, showing that metabolism powers mobility within the highly viscous bacterial cytoplasm [Parry et al., 2014]. Importantly, these studies indicate that the limits of particle mobility (proteins, mRNAs, nucleoprotein complexes) play a crucial role in bacterial physiology.

The speed of translation is defined by the ON-rate − and OFF-rate − kinetics of ribosome-binding factors. A theoretical study has shown that translation operates close to the limit of diffusion, set by macromolecular crowding in cells and that most likely, diffusion of EF-Tu/charged tRNA complexes (ternary complex, TC) is the rate-limiting factor [Klumpp et al., 2013]. Likely to compensate for this, the four EF-Tu-binding sites on the ribosome are saturated with EF-Tu/TCs, generating a high local concentration that may greatly enhance the rate of testing of aa-tRNAs at the anticodon site, as a recent SMT study has shown [Mustafi and Weisshaar, 2018]. While EF-Tu is in up to 10-fold excess over ribosomes, 60% of molecules were found in a low-mobility mode, likely bound to ribosomes, and only 40% appeared to diffuse freely through the cell [Mustafi and Weisshaar, 2018, 2019]. Accumulation of translating ribosomes at polar regions may additionally counteract the limit for diffusion, which might be also the case for metabolic enzymes (see above).

Fast-growing cells employ almost half of their translation capacity to synthesize new ribosomes, and, as explained above, ribosomal protein synthesis occurs in clusters at subpolar regions of cells with nucleoid-type organization [Jin et al., 2017]. RNAP forms large clusters at origin regions, especially during fast growth. These nucleoid substructures also contain accumulations of NusB and NusG, two antitermination proteins essential for efficient transcription of rRNA genes [Doherty et al., 2006], which are full of termination sites. It therefore seems appropriate to term these assemblies “nucleolus-like” structures. Interestingly, if all chromosomal rRNA operons are deleted in *E*. *coli* cells, and a single operon is placed on a plasmid, the plasmids localize to the polar boundary of the nucleoids, recruit an RNAP cluster, and also induce the accumulation of NusB [Mata Martin et al., 2018]. Thus, an rRNA operon can lead to the formation of a nucleolus-like assembly *in trans*. It seems very handy that clustered transcription of ribosomal RNA is located at the outer borders of nucleoids, directly adjacent to sites of ribosomal protein synthesis (Fig. 1), setting up short distances for diffusion, if not direct coupling of rRNA synthesis with ribosome maturation. We speculate that GTPases that are essentially involved in ribosome assembly [Britton, 2009] are also recruited to subpolar sites and do not predominantly diffuse freely throughout the cells, such that ribosome assembly follows the logic of a pseudo-nucleolus, in addition to the pseudo-nucleus arrangement in cells containing nucleoids.

## Conclusions

Bacterial cells appear to harvest entropic effects, inter- and intramolecular attraction and repulsion, and size-dependent volume exclusion for optimal subcellular organization of DNA metabolism, translation, and general metabolism. In recent years, liquid-liquid phase separation has been identified as an additional contributor to intracellular organization [Shin and Brangwynne, 2017]. The resulting liquid-phase protein condensates could be described as membrane-less organelles, thought to also exist in prokaryotic cells. For example, RNAPs appear to rapidly form such clusters during exponential growth in *E*. *coli*. By utilizing single molecule tracking, it was identified that molecules inside the clusters are dynamic and move at different speeds than on the outside [Ladouceur et al., 2020]. Future challenges will be to investigate how protein condensates arise on a molecular basis and affect different processes inside the cell, which promises intriguing new insights into intracellular organization.

## Conflict of Interests Statement

The authors declare no conflict of interest.

## Funding Sources

Work in the author's laboratory was funded by the LOEWE program from the State of Hessen, by the Bundesministerium für Bildung und Forschung (BMBF, funding for program NANOKAT) and by the Deutsche Forschungsgemeinschaft. The funders have not played any role in the design or interpretation if this work.

## Author Contributions

Simon Dersch has written the manuscript and designed Figure 1; Daniel Andreas Orlando Rotter has helped write the manuscript and has designed Figures 2 and 3; and Peter L. Graumann has written the manuscript and obtained funding for the work.
